# Supplementary material for: Novel terpolymers based on methyl methacrylate with superior thermal stability and optical transparency for high-value applications
Source: PLoS One. 2025 Oct 8;20(10):e0332300. doi: 10.1371/journal.pone.0332300 (PMC12507251; doi:10.1371/journal.pone.0332300)
Supplement: S1 File — (ZIP) [file pone.0332300.s001.zip › Supporting_Information/Table 1 and Table 2/Table 1 and Table 2 .docx]

**Table 1. Elemental Analysis (CHN) of the P(MMA40-VBC30-MI30) Terpolymer.**

| **Sample** | **Experimental Value (%)** | | | **Theoretical Value (%)** | | | **Difference (%)** | | |
| --- | --- | --- | --- | --- | --- | --- | --- | --- | --- |
|  | **N%** | **C%** | **H%** | **N%** | **C%** | **H%** | **N%** | **C%** | **H%** |
|  | 2.7 | 59.3 | 6.9 | 5.4 | 61.79 | 5.34 | 50 | 4.03 | -29.21 |

**Table 2. Thermal Properties and Monomer Composition of Synthesized Polymers.**

| **Polymer Composition** | **Tg (°C)** | **Char Yield (%)** | **TMax (°C)** | **T50 (°C)** | **T10 (°C)** | **MI%** | **VBC%** | **MMA%** |
| --- | --- | --- | --- | --- | --- | --- | --- | --- |
| PMMA | 104 | 8.0 | 371 | 351 | 221 | 0 | 0 | 100 |
| P(MMA) | 115 | 18.98 | 514 | 508 | 454 | 0 | 100 | 0 |
| P(MMA50-VBC50) | 119 | 0 | 372 | 352 | 255 | 0 | 50 | 50 |
| P(MMA60-VBC20-MI30) | 119 | 10 | 335 | 338 | 270 | 30 | 10 | 60 |
| P(MMA50-VBC20-MI30) | 119 | 12 | 336 | 347 | 309 | 30 | 20 | 50 |
| P(MMA40-VBC30-MI30) | 119 | 14 | 336 | 353 | 319 | 30 | 30 | 40 |
