# Supplementary figures and images for: Novel terpolymers based on methyl methacrylate with superior thermal stability and optical transparency for high-value applications
Source: PLoS One. 2025 Oct 8;20(10):e0332300. doi: 10.1371/journal.pone.0332300 (PMC12507251; doi:10.1371/journal.pone.0332300)

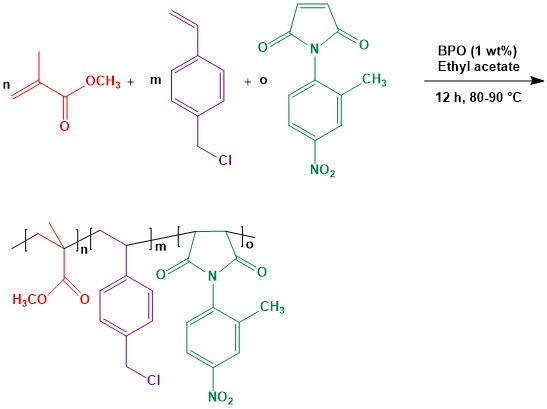

Supplement: S1 File — (ZIP) [file pone.0332300.s001.zip › Supporting_Information/Fig. 1. Reaction scheme. Radical polymerization reaction of the terpolymer (methyl methacrylate vinylbe.tif/Fig. 1. Reaction scheme. Radical polymerization reaction of the terpolymer (methyl methacrylate vinylbe.tif]

PMMA


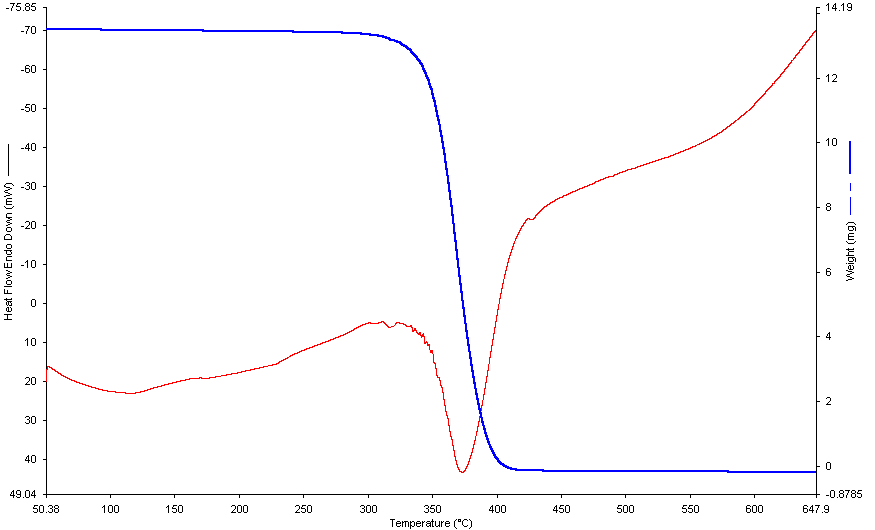


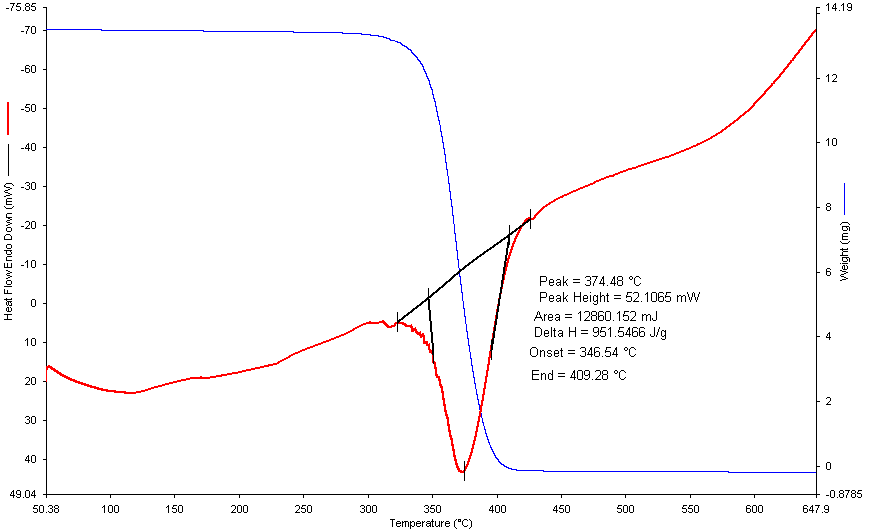


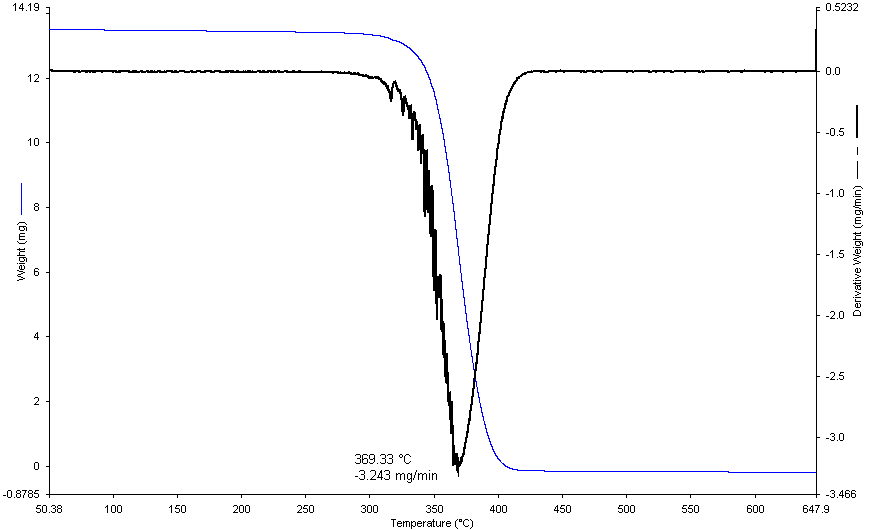


10%


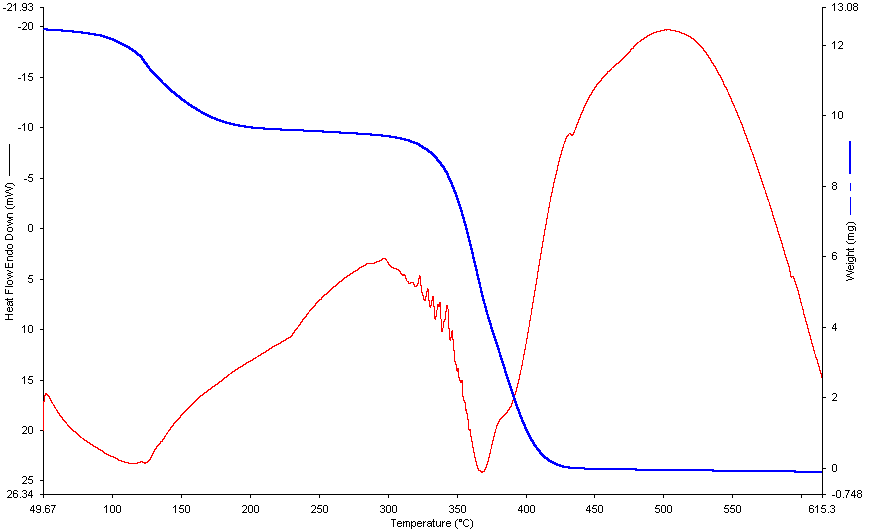


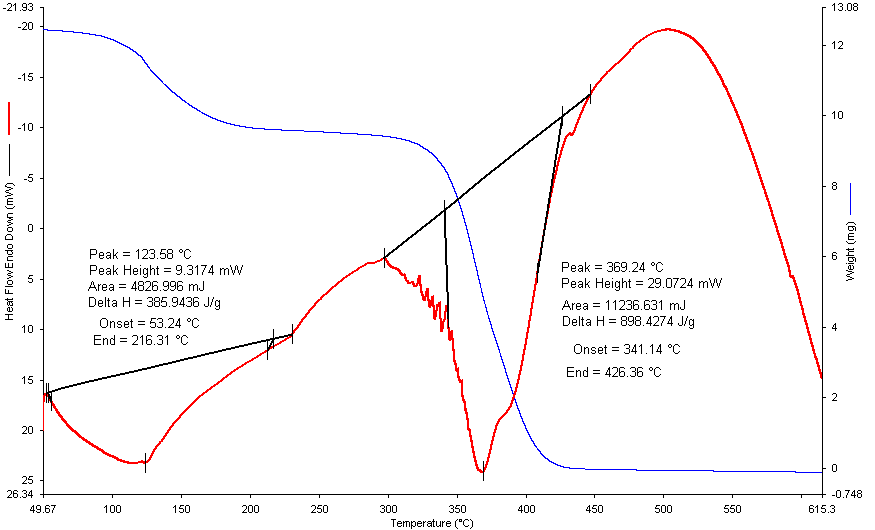


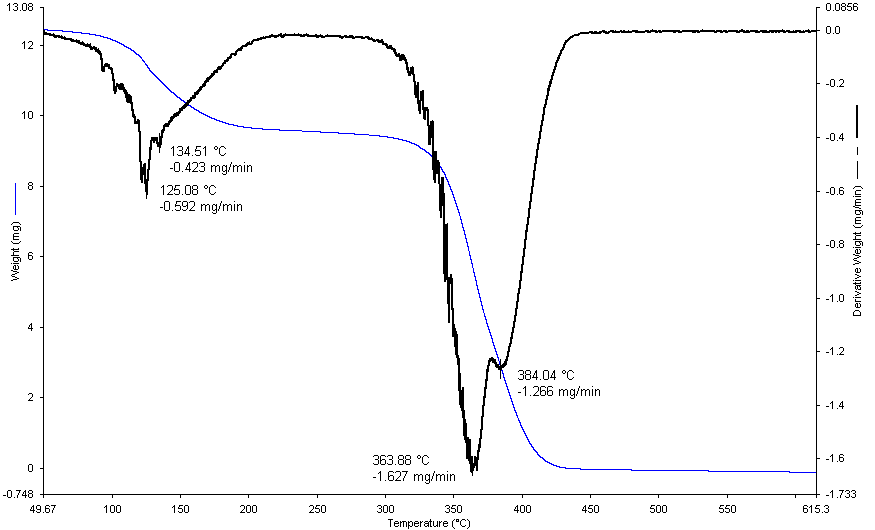


20%


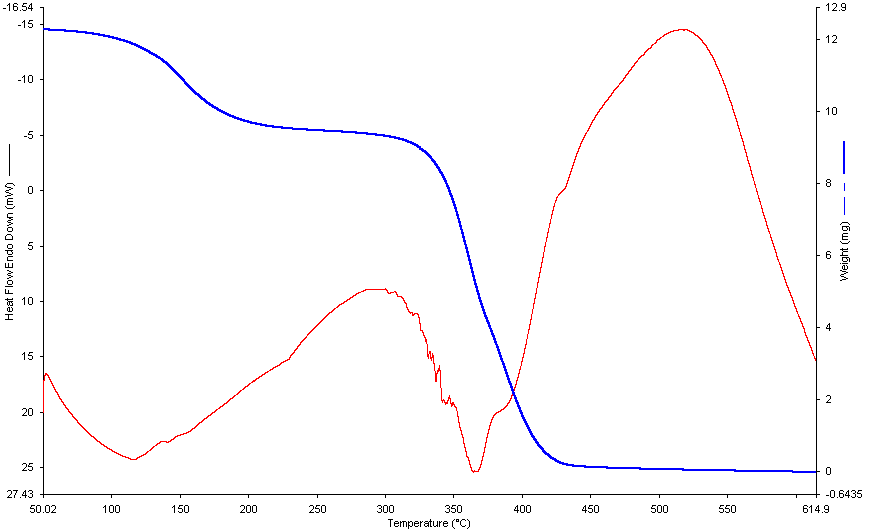


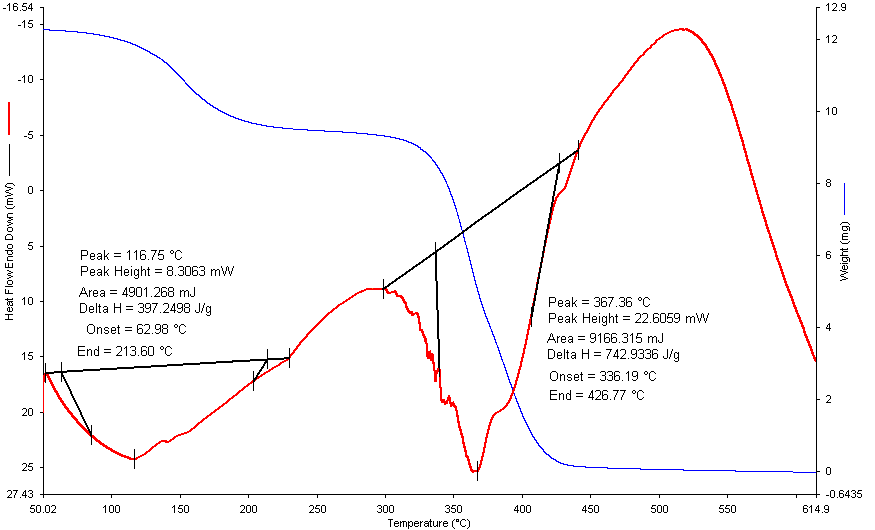


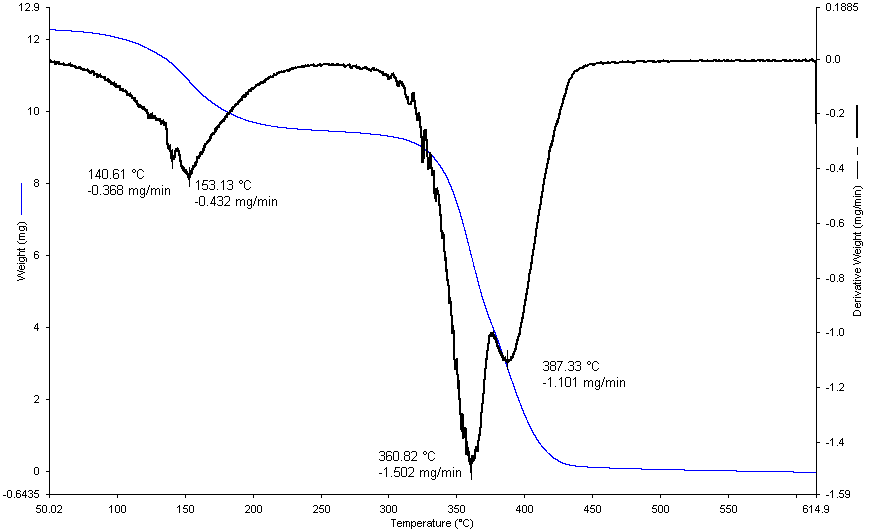


30%


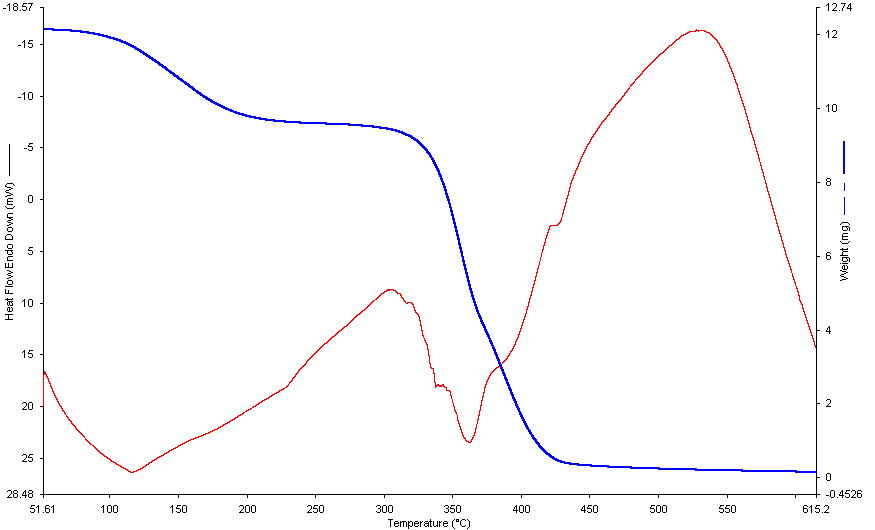


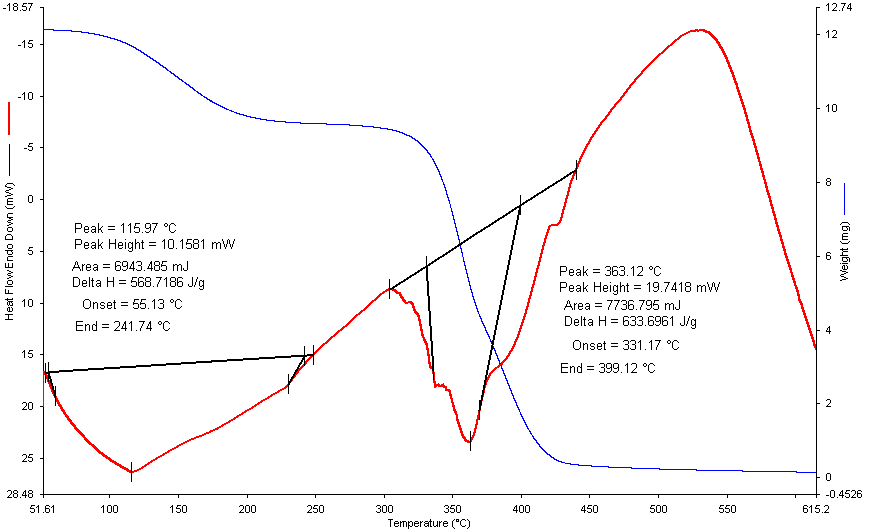


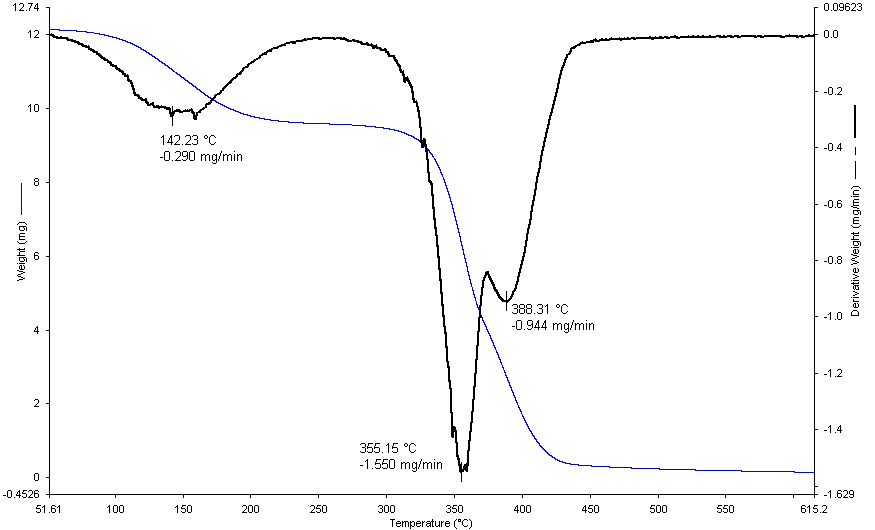

Supplement: S1 File — (ZIP) [file pone.0332300.s001.zip › Supporting_Information/Fig. 5. Derivative thermogravimetric (DTG) curves comparing the thermal degradation rates of the synthesized/TGA-DSC/DTG (PMMMA)/PMMA,(10.20.30)%.docx]

VBC


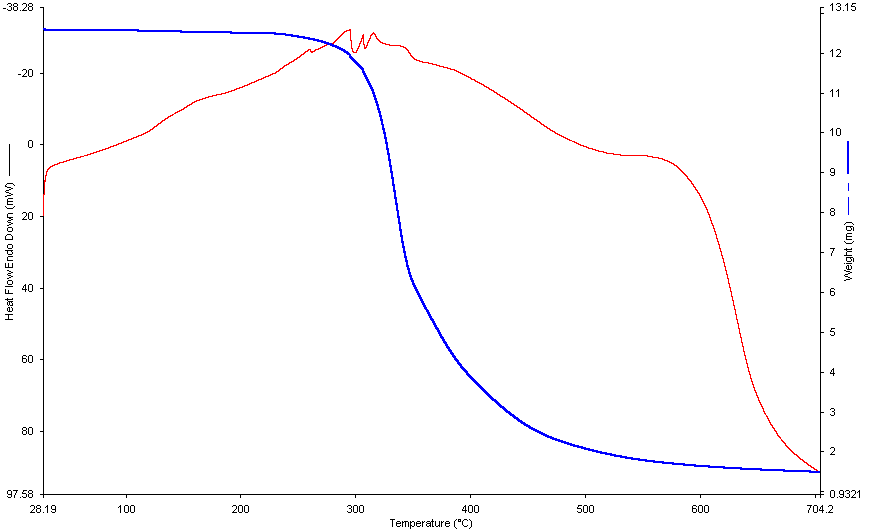


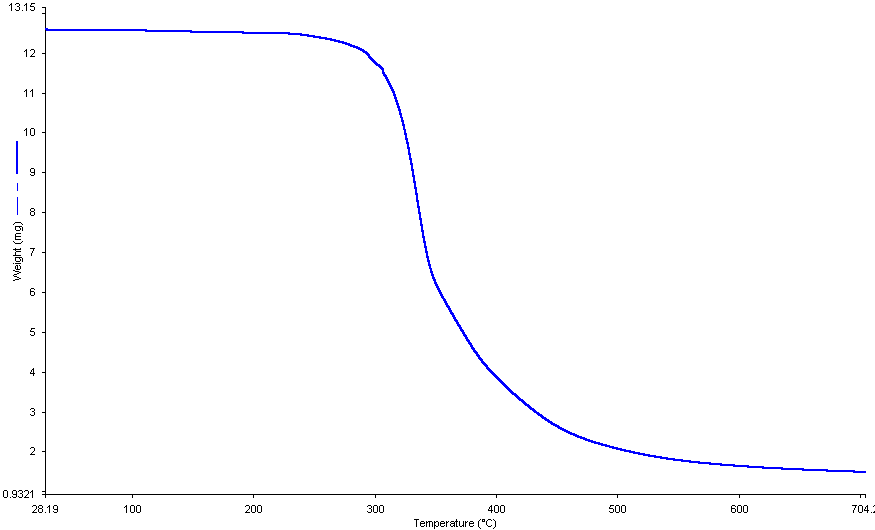


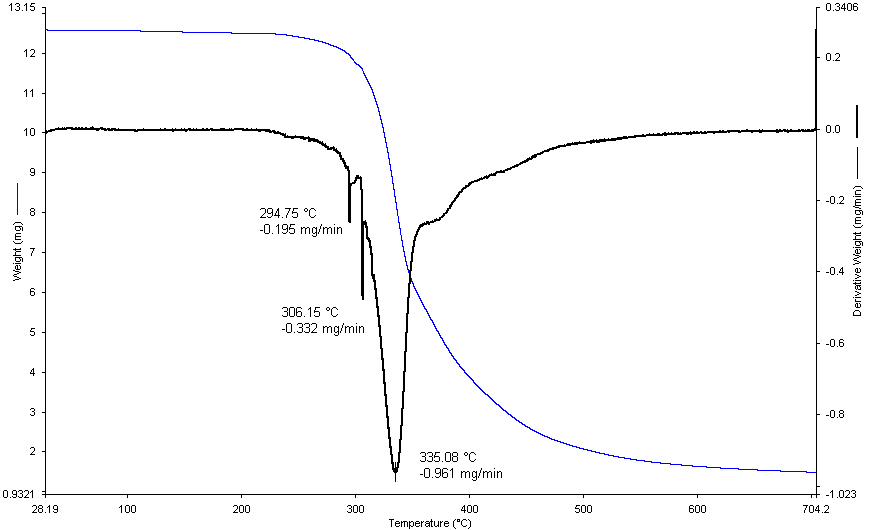

Supplement: S1 File — (ZIP) [file pone.0332300.s001.zip › Supporting_Information/Fig. 5. Derivative thermogravimetric (DTG) curves comparing the thermal degradation rates of the synthesized/TGA-DSC/DTG(poly(MMA(40)-VBC(30)-MI(30))/VBC.docx]

Poly VBC


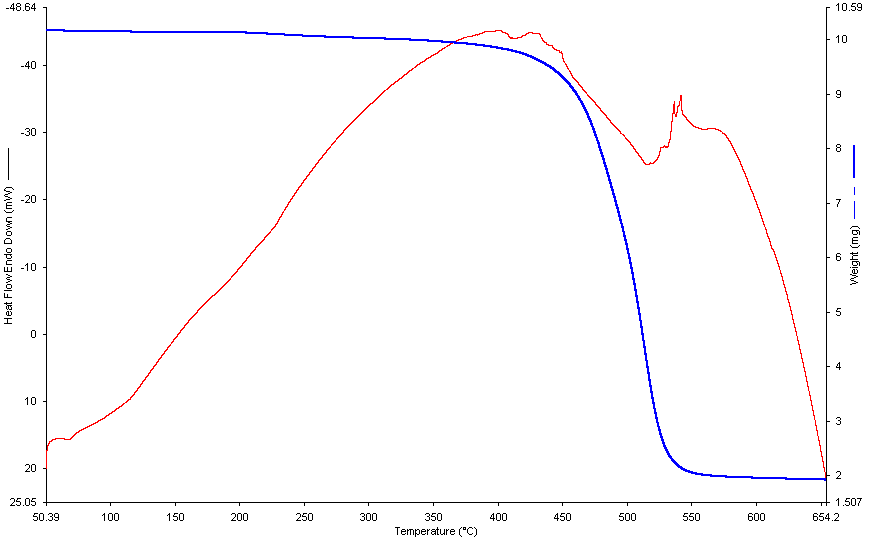


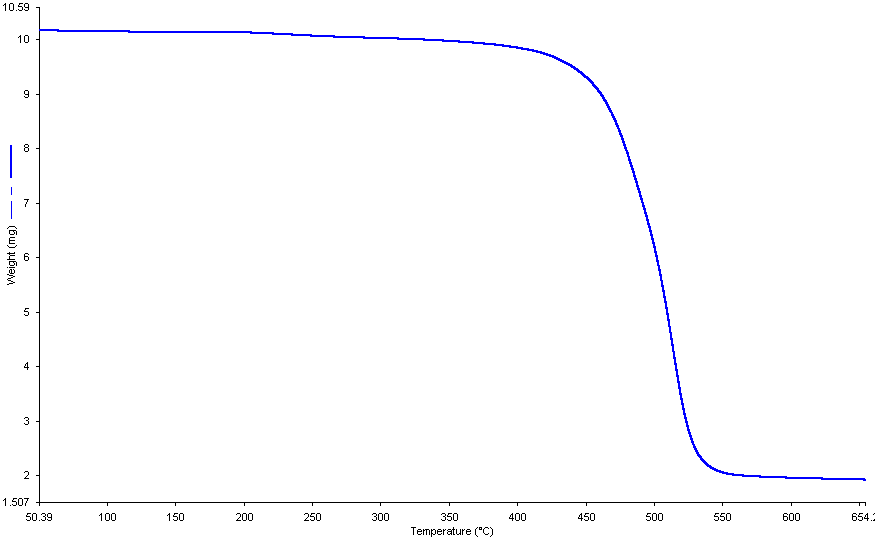


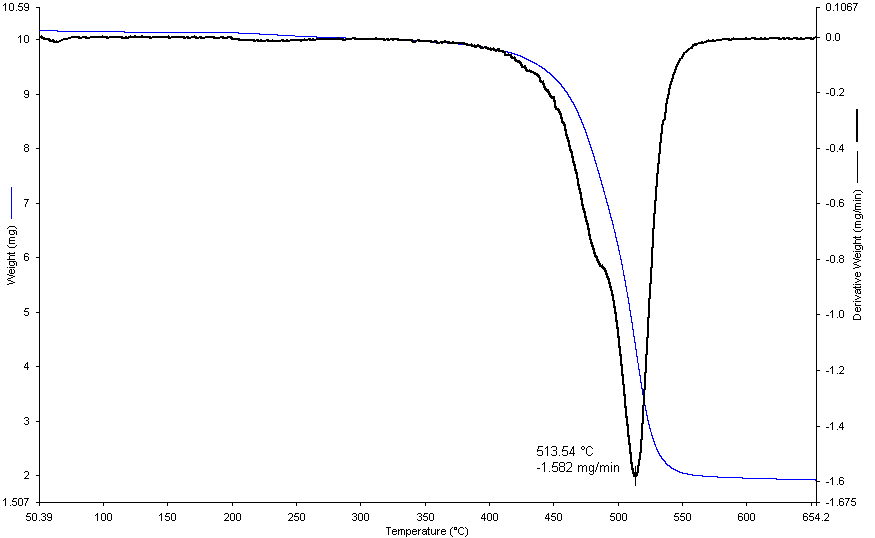


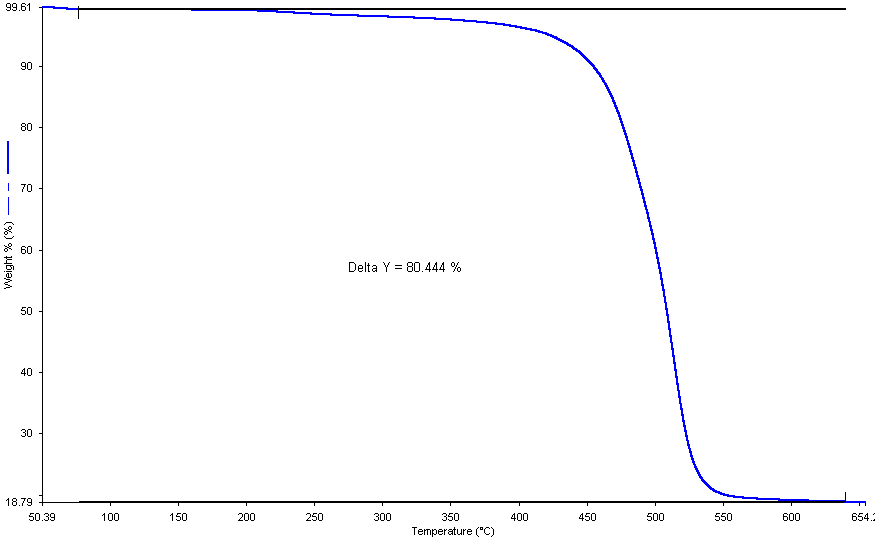

Supplement: S1 File — (ZIP) [file pone.0332300.s001.zip › Supporting_Information/Fig. 5. Derivative thermogravimetric (DTG) curves comparing the thermal degradation rates of the synthesized/TGA-DSC/DTG(poly(VBC)/Poly VBC.docx]

TVBC 10%


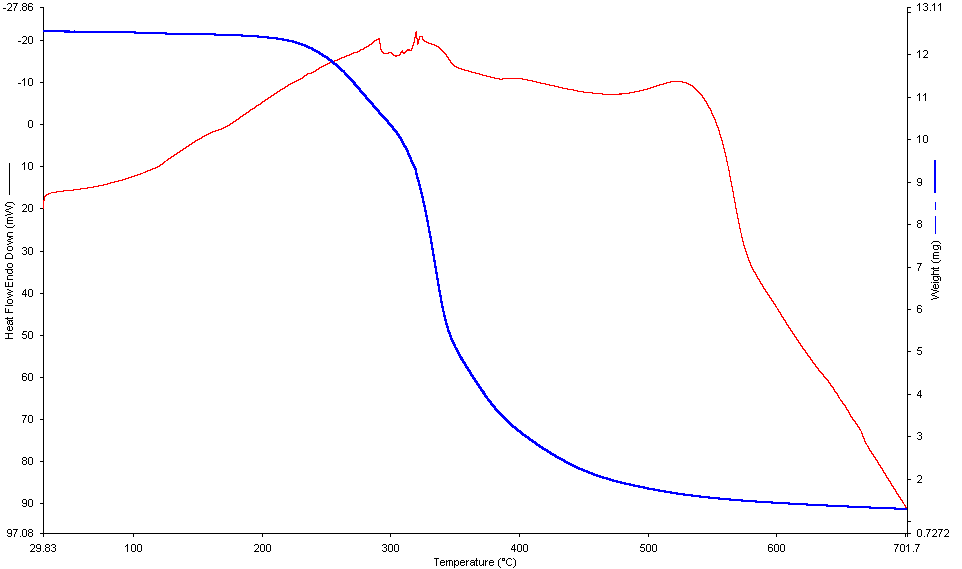


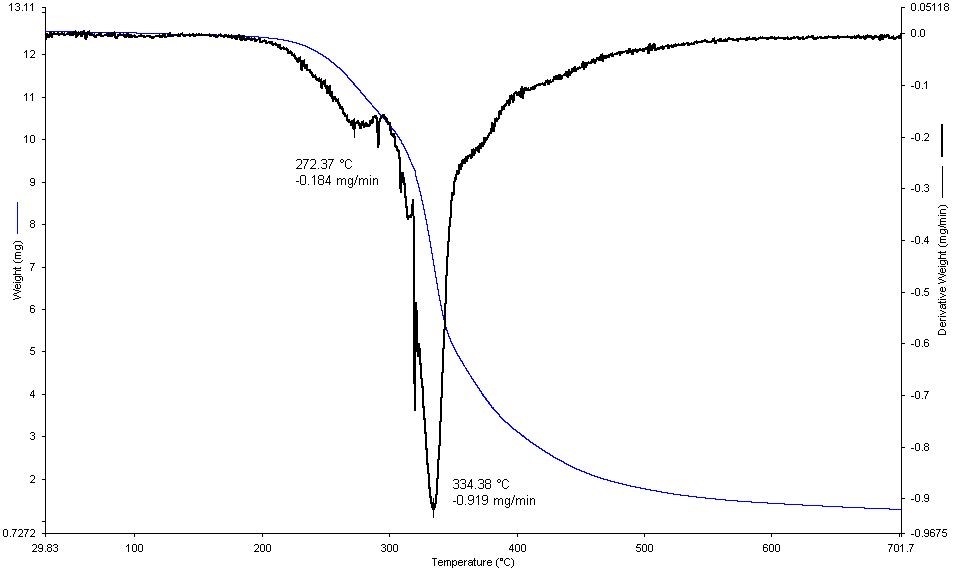


VBC 20%


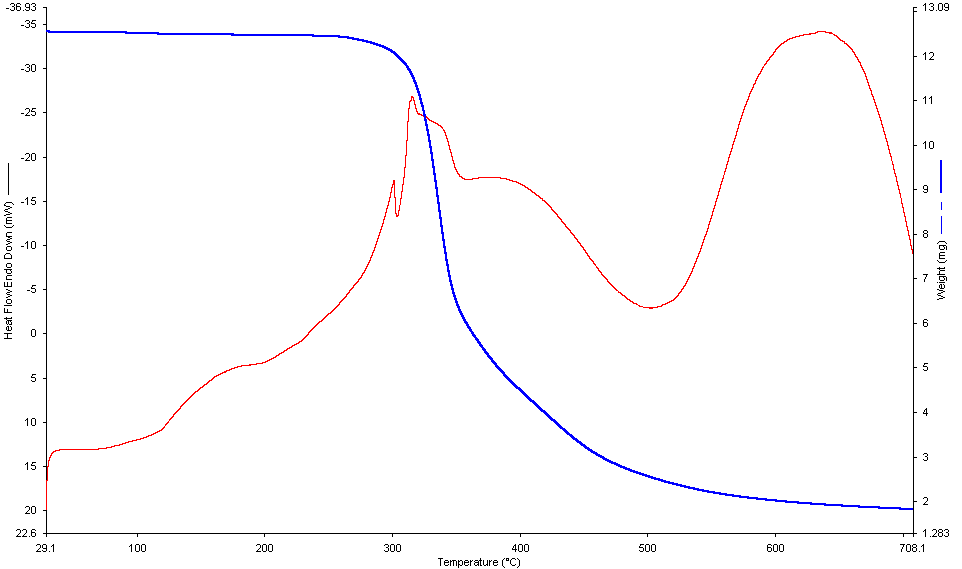


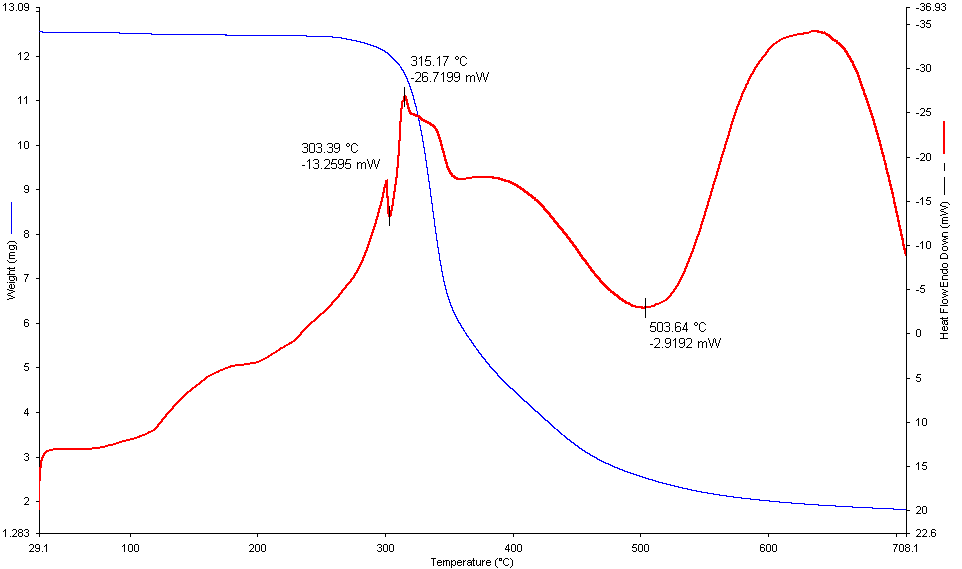


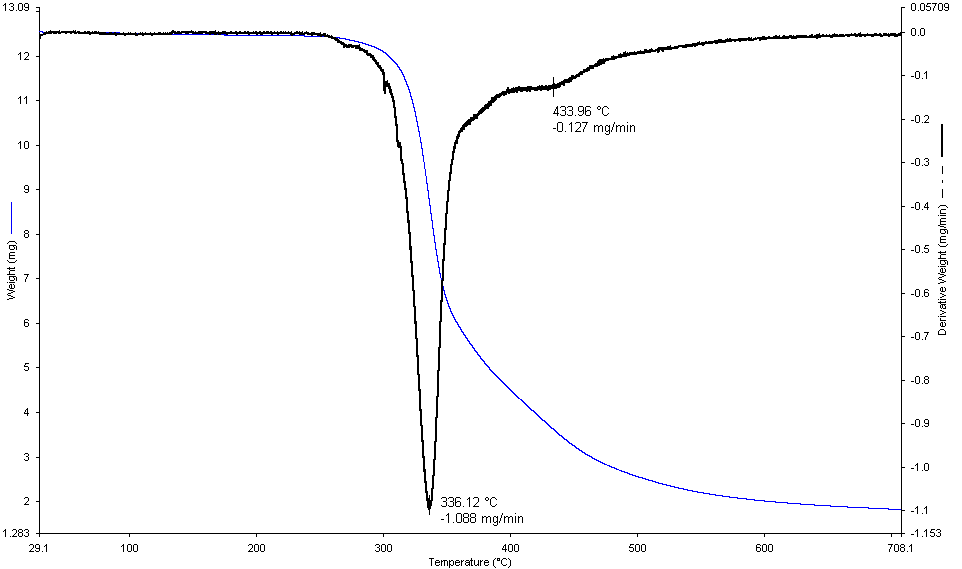


VBC .MMA(50.50)


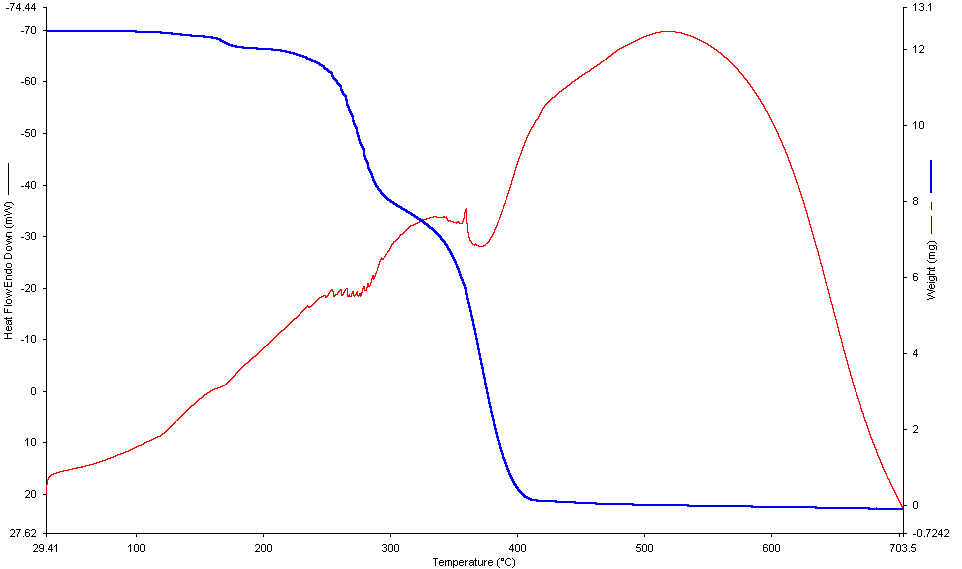


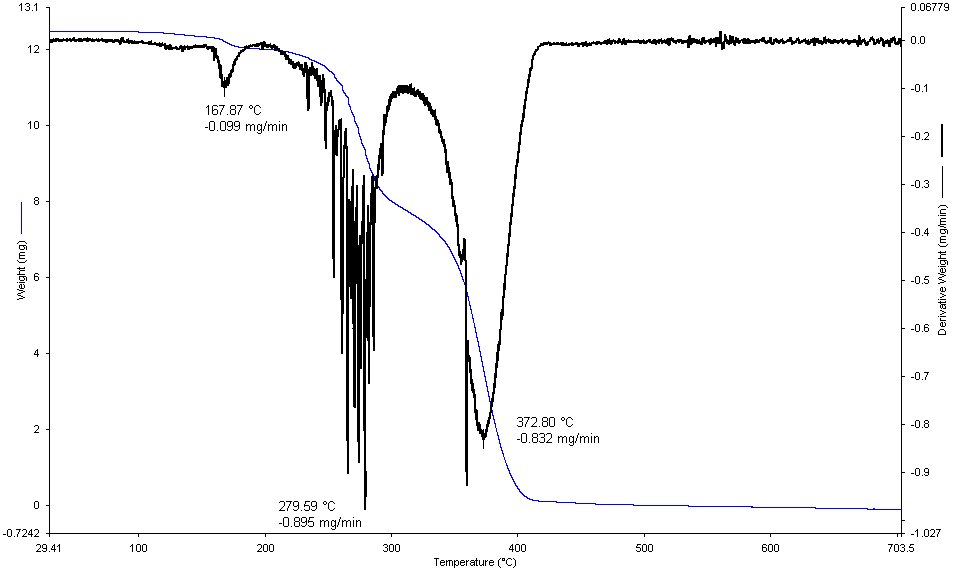

Supplement: S1 File — (ZIP) [file pone.0332300.s001.zip › Supporting_Information/Fig. 5. Derivative thermogravimetric (DTG) curves comparing the thermal degradation rates of the synthesized/TGA-DSC/DTG(poly(VBC)/VBC samples.docx]
