# Supplementary material for: Novel terpolymers based on methyl methacrylate with superior thermal stability and optical transparency for high-value applications
Source: PLoS One. 2025 Oct 8;20(10):e0332300. doi: 10.1371/journal.pone.0332300 (PMC12507251; doi:10.1371/journal.pone.0332300)
Supplement: Attachment — Submitted filename: Response to Reviewers.docx [file pone.0332300.s002.docx]

Dear Dr. Nayan Ranjan Singha, Academic Editor, and Reviewers,

Thank you for the detailed review and constructive comments on our manuscript entitled "**Novel Terpolymers Based on Methyl Methacrylate with Superior Thermal Stability and Optical Transparency for High-Value Applications**". We are grateful for the opportunity to revise our manuscript. We have carefully considered all the points raised and have revised the manuscript accordingly.

**To facilitate the review process, all changes made in the revised manuscript have been highlighted in blue.**

Our point-by-point responses to the comments are provided below.

**Comments from Reviewer #1**

**Comment 1:** Modify the title, avoid abbreviation from from the title.

**Response:** We thank the reviewer for this constructive suggestion. We have modified the title to avoid abbreviations and to enhance clarity. The new title is: "Novel Terpolymers Based on Methyl Methacrylate with Superior Thermal Stability and Optical Transparency for High-Value Applications."

**Comment 2:** In the abstract: Include specific quantitative results to highlight key findings, simplify complex sentences, and ensure a logical flow from background to conclusion.

**Response:** We thank the reviewer for this excellent suggestion. We have thoroughly revised the abstract to include specific quantitative results that highlight the key findings, such as the increase in T10 (from 221°C to 319°C), Tg (from 104°C to 119°C), and light transmission (from 90% to 100%). We have also simplified complex sentences and restructured the abstract to ensure a clear and logical flow, moving from the background and problem statement to the key findings and final conclusion.

**Comment 3:** Every abbreviation should be expanded and then give its short form in the entire the manuscript

**Response:** We thank the reviewer for this helpful comment. We have reviewed the entire manuscript to ensure that every abbreviation is expanded at its first appearance, followed by its short form in parentheses. This standard has been consistently applied throughout the text for clarity.

**Comment 4:** Revise the graphical abstract.

**Response:** We thank the reviewer for this suggestion. We have thoroughly revised the graphical abstract to more clearly and effectively depict the key findings of our study, particularly the synergistic improvement in thermal stability and optical transparency. The new graphical abstract visually summarizes the synthesis process and the remarkable enhancement of the final terpolymer's properties.

**Comment 5:** The introduction is part is too long with 1100 words. The introduction gives a thorough overview of PMMA’s industrial importance, limitations, and previous modification strategies, clearly identifying the gap in synergistically incorporating VBC and MI to enhance both thermal stability and optical clarity. However, the section could be improved by condensing repeated points on PMMA’s limitations and prior aromatic comonomer studies, shortening long sentences for clarity, highlighting the novelty earlier to engage readers before the detailed literature review, and correcting minor typographical and reference formatting issues.

**Response:** We thank the reviewer for their detailed and helpful comments on our Introduction section. We have thoroughly revised the introduction to address all the points raised. The revised version has been condensed by removing repeated points and restructuring the content for a more logical and concise flow. We have also shortened several long sentences to improve readability and clarity. The novelty and the specific research gap our study addresses have now been highlighted earlier in the text to better engage the reader. Finally, we have carefully proofread the entire section to correct any minor typographical and reference formatting issues.

**Comment 6:** The author should provide all chemicals used in the study, including their sources and purities. It is crucial to specify the purity of the raw materials, as the accuracy and reliability of the results depend significantly on the quality of the samples used.

**Response:** We thank the reviewer for this valuable comment. We have revised the "Materials" section to provide a comprehensive list of all chemicals used in the study. We have included the name of the supplier and the purity grade for each chemical. To ensure the accuracy and reliability of our results, we also specified which materials underwent further purification and detailed the purification methods. The revised section now fully addresses the reviewer's request.

**Comment 7:** The heating rate used for the thermal properties (DSC and TGA) is missing in section 2.4.

**Response:** We thank the reviewer for pointing this out. The heating rate for the thermal properties analysis has been specified in the "Instrument and Measurement" section as follows: "at a heating rate of 5 °C/min".

**Comment 8:** Avoid bulk references in the discussion section, and instead support each key finding with specific, relevant literature citations.

**Response:** We thank the reviewer for this important and insightful comment. We have thoroughly revised the Discussion section to replace all bulk references with specific, relevant literature citations. Each key finding, claim, and data point that is supported by external literature is now referenced immediately, ensuring a more precise and academically rigorous presentation.

**Comment 9:** Thermal Properties: Enhanced Stability from Aromatic Incorporation. Need more reference in this section.

**Response:** We thank the reviewer for this constructive suggestion. We have revised the "Thermal Properties: Enhanced Stability from Aromatic Incorporation" section and added more relevant literature citations to better support our findings and provide a more comprehensive discussion on the enhanced thermal stability of the synthesized polymers.

**Comment 10:** All subfigures in a given figure need to be labeled and explained in the figure title.

**Response:** We thank the reviewer for this constructive comment. We have revised our figures to ensure that all subfigures are clearly labeled (e.g., a, b, c) and that the corresponding figure title provides a complete and detailed explanation of each label.

**Comment 11:** Compare the results obtained in this study can be compared with other results and hence to point out the advancement of this work.

**Response:** We thank the reviewer for this constructive suggestion. We have added a dedicated section titled "Broader Implications and Comparison with Literature" to explicitly compare our findings with existing studies. This section highlights the advancements of our work, particularly in demonstrating a synergistic effect that successfully enhances both thermal stability and optical clarity, thereby overcoming the trade-offs often reported in previous research.

**Comment 12:** The conclusion part is too long. The Conclusion should illustrate the advances and claims of innovative aspects of the research work done.

**Response:** We thank the reviewer for this constructive comment. We have thoroughly revised the Conclusion section to make it more concise and focused. The new conclusion now clearly highlights the key advances and innovative aspects of our research, including the successful co-optimization of thermal stability and optical transparency, and its broader implications for designing high-performance polymer materials.

**Comment 13:** There are few syntax errors in this manuscript

**Response:** We thank the reviewer for their careful review and for highlighting these points. The entire manuscript has been thoroughly proofread and edited to correct all syntax, grammar, and typographical errors.

**Comments from Reviewer #2**

**Comment 1:** The molecular weights and dispersity values of the copolymers should be determined. Molecular weights of products should be obtained by using GPC.

**Response:** We appreciate the reviewer's valuable recommendation to include Gel Permeation Chromatography (GPC) data for direct molecular weight determination. We fully acknowledge the importance of GPC in comprehensively characterizing polymer molecular weight distribution, which is indeed critical for reproducibility and a deeper understanding of polymer properties.

However, during the period of this study, we faced limitations in access to GPC instrumentation, which regrettably prevented us from performing these measurements. Our use of viscosity measurements as a robust indicator of molecular weight trends aligns with similar approaches in the literature, particularly in studies focused on polymer property correlations. **For instance, a study by Atabaki et al. (2016) also used viscometric analysis as a key method for polymer characterization, which is consistent with our approach.**

To address the aspect of molecular weight characterization, we thoroughly investigated viscosity measurements as a robust indirect indicator of molecular weight trends within our synthesized polymers. As detailed in the "Viscosity Measurements: Indicating Molecular Weight Trends" section of our manuscript, we extensively measured the relative viscosity (ηr) and intrinsic viscosity (ηinh) of all synthesized polymers. We believe that the observed variations in intrinsic viscosity, which directly correlate with changes in polymer chain dimensions and, consequently, molecular weight under consistent experimental conditions, provide a reliable assessment of the relative molecular weight differences and trends among the homopolymer, copolymer, and terpolymers. This approach offered sufficient insights into the polymerization process and the structural characteristics influencing the observed improvements in thermal and optical properties within the scope of our current study.

We are committed to incorporating GPC analysis in our future research endeavors to further complement these findings.

**Comment 2:** In DSC termograms, which direction 'endo' or 'exo' is?

**Response:** We thank the reviewer for this important clarification. We have updated the manuscript to explicitly state the direction of endothermic and exothermic events in the DSC thermograms. As noted in the "Results and Discussion" section and the caption of Figure 6, an endothermic event is represented by a positive deflection (upward direction).

**Reviewer #2, Comment 3**

**Comment3:** The Conclusions section is too long. The Conclusions section should be revised. The Conclusion section should be clear.

**Response:** We thank the reviewer for this constructive comment. We have thoroughly revised the Conclusion section to make it more concise and focused. The new conclusion now clearly highlights the key advances and innovative aspects of our research, including the successful co-optimization of thermal stability and optical transparency, and its broader implications for designing high-performance polymer materials.

**Comment 4:** The subject is an interesting area. The manuscript probably contained novel data, but the information given is insufficient to estimate its reliability. The introduction is insufficient. It should be rewritten with clear reasoning justifying publication. The following references related to copolymerization of MMA should be added in the introduction part for a good understanding: i) Journal of Chemical Sciences 136, 68 (2024) ii) Chemical Papers 76, 869–878 (2022) iii) Journal of Macromolecular Science Part A 57(12), 819–825 (2020).

**Response:** We thank the reviewer for their valuable and insightful comments regarding the Introduction. We have thoroughly revised the Introduction to address the points raised. The revised Introduction now presents a clear argument for the novelty and significance of our research by highlighting a critical gap in the existing literature. We have also incorporated the three suggested references to provide a more comprehensive and robust foundation for our study.

**Comment 5:** The Keywords should be at most 5. Reduce the number of keywords.

**Response:** We thank the reviewer for this helpful reminder. We have revised our list of keywords and reduced the number of keywords to a maximum of five, as suggested.

**Comment 6:** PLOS authors have the option to publish the peer review history of their article (what does this mean?). If published, this will include your full peer review and any attached files. If you choose “no”, your identity will remain anonymous but your review may still be made public. Do you want your identity to be public for this peer review?

**Response:** We thank the reviewer for this question. After careful consideration, we have decided not to publish the peer review history of our article. We appreciate the time and effort the reviewers dedicated to improving our manuscript
